# Identification of biomarkers associated with metabolic cardiovascular disease using mRNA-SNP-miRNA regulatory network analysis

**DOI:** 10.1186/s12872-021-02166-4

**Published:** 2021-07-23

**Authors:** Zhiyuan Fan, Wenjuan Peng, Zhiwen Wang, Ling Zhang, Kuo Liu

**Affiliations:** 1 Department of Epidemiology and Health Statistics, School of Public Health, Capital Medical University, and Beijing Municipal Key Laboratory of Clinical Epidemiology, Beijing, China; 2 Department of Urology, Beijing Friendship Hospital, Capital Medical University, Beijing, China

**Keywords:** CVD, T2DM, mRNA, SNP, miRNA, Interaction network

## Abstract

**Background:**

CVD is the leading cause of death in T2DM patients. However, few biomarkers have been identified to detect and diagnose CVD in the early stage of T2DM. The aim of our study was to identify the important mRNAs, micro (mi)RNAs and SNPs (single nucleotide polymorphisms) that are associated with metabolic cardiovascular disease.

**Materials and methods:**

Expression profiles and GWAS data were obtained from Gene Expression Omnibus (GEO) database. MiRNA-sequencing was conducted by Illumina HiSeq 2000 platform in T2DM patients and T2DM with CVD patients. EQTL analysis and gene ontology (GO), Kyoto Encyclopedia of Genes and Genomes (KEGG) pathway enrichment analyses were conducted. MRNA-miRNA co-expression network and mRNA-SNP-miRNA interaction network were established and visualized by Cytoscape 3.7.2.

**Results:**

In our study, we identified 56 genes and 16 miRNAs that were significantly differentially expressed. KEGG analyses results indicated that B cell receptor signaling pathway and hematopoietic cell lineage were included in the biological functions of differentially expressed genes. MRNA-miRNA co-expression network and mRNA-SNP-miRNA interaction network illustrated that let-7i-5p, *RASGRP3*, *KRT1* and *CEP41* may be potential biomarkers for the early detection and diagnosis of CVD in T2DM patients.

**Conclusion:**

Our results suggested that downregulated let-7i-5p, and upregulated *RASGRP3*, *KRT1* and *CEP41* may play crucial roles in molecular mechanisms underlying the initiation and development of CVD in T2DM patients.

**Supplementary Information:**

The online version contains supplementary material available at 10.1186/s12872-021-02166-4.

## Background

It is estimated that in 2019 463 million adults aged 20–79 years would develop diabetes with type 2 diabetes (T2DM) accounting for > 90% of cases [1]. The incidence of T2DM has greatly increased in recent years and has become a great threat to human health worldwide. T2DM is one of major risk factors of cardiovascular diseases (CVD), but its mechanism of action is not fully understood [2]. Even newly diagnosed diabetics were reported to have at least one vascular complication, and CVD is the leading cause of death in T2DM patients [3, 4]. Furthermore, people with T2DM are 2–6 times more likely to die of CVD than those without diabetes [5]. However, few biomarkers have been identified to diagnose CVD at the early stage of T2DM.

MicroRNAs (miRNAs) bind to complementary sequences of their target mRNA by base pairing to induce mRNA degradation and/or inhibit translation, thus affecting gene expression after transcription [6]. MiRNA has been found to have high specificity for disease status and may be used as a potential biomarker to predict disease progression. In recent years, multiple miRNAs have been demonstrated to be involved in angiogenesis and endothelial cells dysfunction [7–9]. Expression of miRNAs can be affected by many factors. The miRNA seed region can specifically bind to miRNA recognition element (MRE) in the 3′UTR (3′ untranslated region) of their target mRNA [10]. Therefore, any anomalies of miRNA-MRE interaction may have an impact on gene expression and lead to diseases [11–13]. Single nucleotide polymorphism (SNP), the most common genetic variants, can interfere with the base pairing between miRNA and its target mRNA, affecting normal expression of genes and eventually contributing to disease pathogenesis [14]. Genome-Wide Association Studies (GWAS) have found that genetic variants play an important role in pathogenesis of CVD [15], and association between some genetic variants and CVD has critical biological significance for T2DM patients [16, 17]. Genome-wide expression quantitative trait locus (eQTL) analysis is an effective method to study the effect of SNPs on gene expression [18, 19]. However, eQTL analysis mainly focuses on mRNA expression and rarely involves miRNA, which leads to incomplete interaction patterns [20].

To more comprehensively reveal the complicated association between mRNA, miRNA and SNP, and to find potential biomarkers with high specificity and sensitivity to diagnose CVD at the early stage of T2DM, we performed an integrative analysis. EQTL analysis and gene ontology (GO), KEGG pathway enrichment analyses were also conducted to better understand the connection between mRNA, miRNA and SNP, and their potential effect on CVD in T2DM patients.

## Materials and methods

### Sample processing and miRNA profiling

Six diabetes patients and five diabetes with ischemic heart disease were recruited from communities in Beijing after informed consent was obtained from each participant. T2DM was diagnosed according to American Diabetes Association Criteria 【24357215】. Ischemic heart disease was defined by clinical history, including acute myocardium infarction, angina pectoris and/or ischemic electrocardiographic alterations. All the participants did not have diabetic retinopathy, nor diabetic microvascular complications. This study was approved by the Ethics Committee of Capital Medical University (No. 2016SY24).

Total RNA extraction was performed using TRIzol (Invitrogen, USA) according to manufacturer’s instructions. After removing DNA contamination by DNase I treatment, total RNA was assessed by NanoDrop spectrophotometer (NanoDrop, USA). A total of 2 μg of RNA/sample was used for the miRNA library. TruSeq Small RNA Sample Preparation Kit (Illumina, Inc., San Diego, CA, USA) was used to generate miRNA sequencing libraries. The library concentration was assessed by Qubit Spectrophotometer and the miRNA sequencing library quality was obtained by using the Agilent 2100 Bioanalyzer system with a High Sensitivity DNA Kit (Agilent Technologies). Sequencing was performed on an Illumina HiSeq 2000 platform and 50 bp of single-end reads were generated. We used fastqc v0.10.1 (http://www.bioinformatics. babraham.ac.uk/projects/fastqc/) to check the quality of raw reads. The reads were mapped to hg19 reference genome to identify mature miRNA, and the expression profile was generated by using miRDeep2 software (https://www.mdc-berlin.de/8551903/en/).

### Microarray datasets and preprocessing

We searched GEO data repository (https://www.ncbi.nlm.nih.gov/geo/) for eligible studies until January 20, 2020. The following terms and different combination of them were used: “type 2 diabetes”, “cardiovascular disease”, “atherosclerosis”, “coronary artery disease”, “heart disease”, “angina pectoris”, “myocardial infarction”, “coronary angiography”, “chronic myocardial ischemia syndrome”, and “acute coronary syndrome”. Based on the search strategy, three datasets were downloaded from GEO, among which GSE90074 and GSE90073 (see Additional file 1: Table S1 for more details) were from same study. Details of each dataset are provided in Table 1.

**Table 1:** Details of microarray datasets from GEO database

GSE	Type	Sample size		Chip
		T2DM	T2DM + CVD	
GSE90074	mRNA	17	38	Agilent-014850 whole human genome microarray 4 × 44 K G4112F
GSE66175	mRNA	48	57	Affymetrix human genome U133A 2.0 array
GSE90073	SNP	13	25	Affymetrix genome-wide human SNP 6.0 array

### Quality evaluation and data preprocessing

The quality of the original CEL files from the dataset GSE66175 (see Additional file 1: Table S1 for more details) was evaluated in this study. We plotted the relative log expression boxplot and normalized unscaled standard errors boxplot to evaluate the consistency of chip quality using affyPLM package (https://git.bioconductor.org/packages/affyPLM/). The degradation of RNA would have a great impact on the quality of chips, thus RNA degradation plot was plotted to show the trend. Then RMA algorithm was used for background correction and normalization of the data. We could obtain the matrix file after quality control and preprocessing in dataset GSE90074, thus we did not do further quality control. Then we converted the probe names from two gene expression profiles into gene names using R 3.6.2 software. And K-Nearest Neighbor in impute package (https://git.bioconductor.org/packages/impute/) was used to fill in the missing value of the gene. Next, we merged two intact gene expression profiles by dplyr package (https://CRAN.R-project.org/package=dplyr). To eliminate difference between batches, we used the combat function in SVA package ((https://bioconductor.org/packages/sva/)) of R 3.6.2 software.

The quality of raw data for miRNA were quality checked by visualization of base quality distributions. To ensure the quality of data analysis, the raw data were filtered and clean data were obtained by using FASTX-Toolkit software (http://hannonlab.cshl.edu/fastx_toolkit/). The following sequences were removed: linker sequence; sequences without 3′ adapter sequences and insert fragments; sequence with Q20 percentage below 60%; sequences outside the length range of 18–36 bp. After the quality control, BLAST alignment (http://blast.ncbi.nlm.nih.gov/) was performed between the miRNA data and the mature miRNA sequences of the corresponding species in the miRBase database (http://www.mirbase.org/), and comparison with the RFAM database (http://rfam.xfam.org/) and the reference genome were performed to conduct a preliminary evaluation of the sequencing results.

### Differential expression analysis

We performed differential expression analysis to find mRNA and miRNA whose |Log twofold change|> 1.5 and *p* value < 0.05 by comparing T2DM patients with T2DM patients complicated with CVD in the R computing environment using limma (https://bioconductor.org/packages/limma/) and DESeq2 packages (https://bioconductor.org/packages/DESeq2/). Next, we plotted volcano plot and heatmap to visualize differentially expressed genes and miRNA using pheatmap package (https://CRAN.R-project.org/package=pheatmap).

### Construction of mRNA-miRNA co-expression network

We performed Pearson correlation analysis to test correlation of differentially expressed mRNA and miRNA. Then, mRNA-miRNA pairs (*p* value < 0.05, absolute value of correlation coefficient > 0.5) were used to construct mRNA-miRNA co-expression network by using software Cytoscape 3.7.2.

### GO and KEGG pathway enrichment analyses

GO and KEGG pathway enrichment analyses were performed to explore the biological process, cellular component, molecular function of differentially expressed genes and other pathways they might get involved in.

### MRNA expression quantitative trait locus analysis

We performed the mRNA expression quantitative trait locus analysis to explore the association between SNP and mRNA expression level by using MatrixEQTL package (https://CRAN.R-project.org/package=MatrixEQTL). Gender was used as a covariate to adjust its effect on mRNA expression. There are two types of eQTL, cis-eQTL which means the difference of gene expression may be caused by the gene itself and trans-eQTL which means the difference of gene expression may be caused by other genes. Then, cis-eQTL and trans-eQTL (*p* value < 0.05) were used for the next analysis.

### Construction of mRNA-SNP-miRNA interaction network

We downloaded SNP data related to differentially expressed miRNA from open sources MirSNP (http://bioinfo.bjmu.edu.cn/mirsnp/search/) and PolymiRTS 3.0 (https://compbio.uthsc.edu/miRSNP/), including SNPs in the binding regions of miRNA and its target mRNA. Next, the SNPs were matched with the cis- and trans- eQTLs of mRNA to find the overlapping SNPs, which could be used to connect mRNA and miRNA. TargetScan (http://www.targetscan.org/mamm_31/) and miRTarBase databases (http://mirtarbase.cuhk.edu.cn/php/index.php) were used to predict the target mRNA of miRNA. Then, we used the overlapping SNPs and the prediction target relationships between mRNAs and miRNAs to construct mRNA-SNP-miRNA interaction networks. Visualization of the mRNA-SNP-miRNA interaction network was performed by Software Cytoscape 3.7.2.

## Results

### Analysis of chip quality

**Fig. 1:**
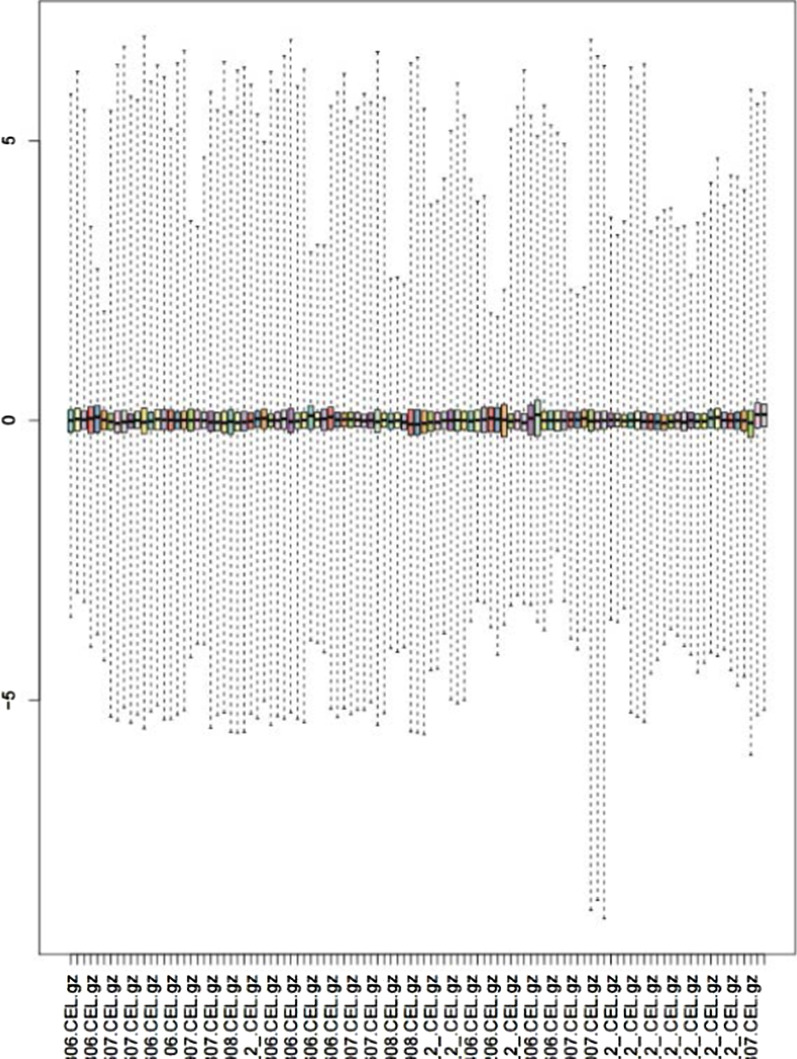
Results from quality evaluation analysis for dataset GSE66175

To ensure the quality of chips included in the study, we evaluated the original CEL files from the dataset GSE66175 to eliminate the chips with poor quality. The relative log expression boxplot showed that the central values of all samples were close to the Y-axis "0" and basically consistent, indicating that all samples were of high quality (Fig. 1). To further evaluate the chip quality, we plotted normalized unscaled standard errors boxplot, in which the standard errors of the samples were all close to the Y-axis "1" with small deviation, indicating that the chip quality was reliable and consistent with the results of the relative log expression boxplot (Additional file 1: Figure S1). The RNA degradation plot showed that the lines of each chip are roughly parallel, indicating that the chip quality is high, which can be used for subsequent data analysis (Additional file 1: Figure S2). We downloaded matrix file of dataset GSE90074 after quality control and preprocessing and integrated it with the dataset GSE66175, then a total of 160 samples (including 65 T2DM patients and 95 T2DM patients with CVD) were included in this study.

**Fig. 2:**
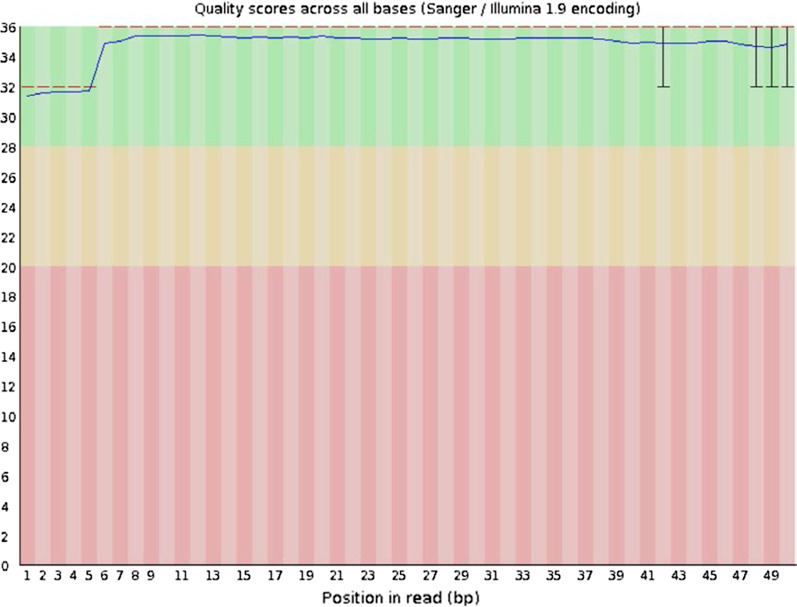
Base quality distributions of raw data

To ensure the quality of data analysis, we visualized the base quality distributions to check the data quality (Fig. 2). In addition, we filtered the raw data and performed data quality control. The quality control results were listed in Table 2.

**Table 2:** Quality control results

Sample ID	*N* after filtration	*N* of high quality	*N* with 18–36 bp	*N* alignments to mature miRNAs in miRBase
DM-01	12,250,616	12,238,294	12,233,798	1530
DM-02	12,281,076	12,268,427	12,264,970	1499
DM-03	10,744,486	10,731,569	10,728,194	1468
DM-04	10,689,640	10,678,807	10,676,732	1440
DM-05	12,995,117	12,981,009	12,975,479	1588
DM-06	9,843,907	9,832,067	9,828,794	1498
DM + CVD-01	13,441,194	13,427,396	13,420,180	1584
DM + CVD-02	15,087,835	15,070,108	15,060,695	1572
DM + CVD-03	12,995,412	12,982,855	12,980,420	1518
DM + CVD-04	9,701,266	9,692,259	9,676,519	1521
DM + CVD-05	11,029,427	11,017,673	11,014,271	1427

*N*: number of sequence

### Differential expression analysis and visualization

**Fig. 3:**
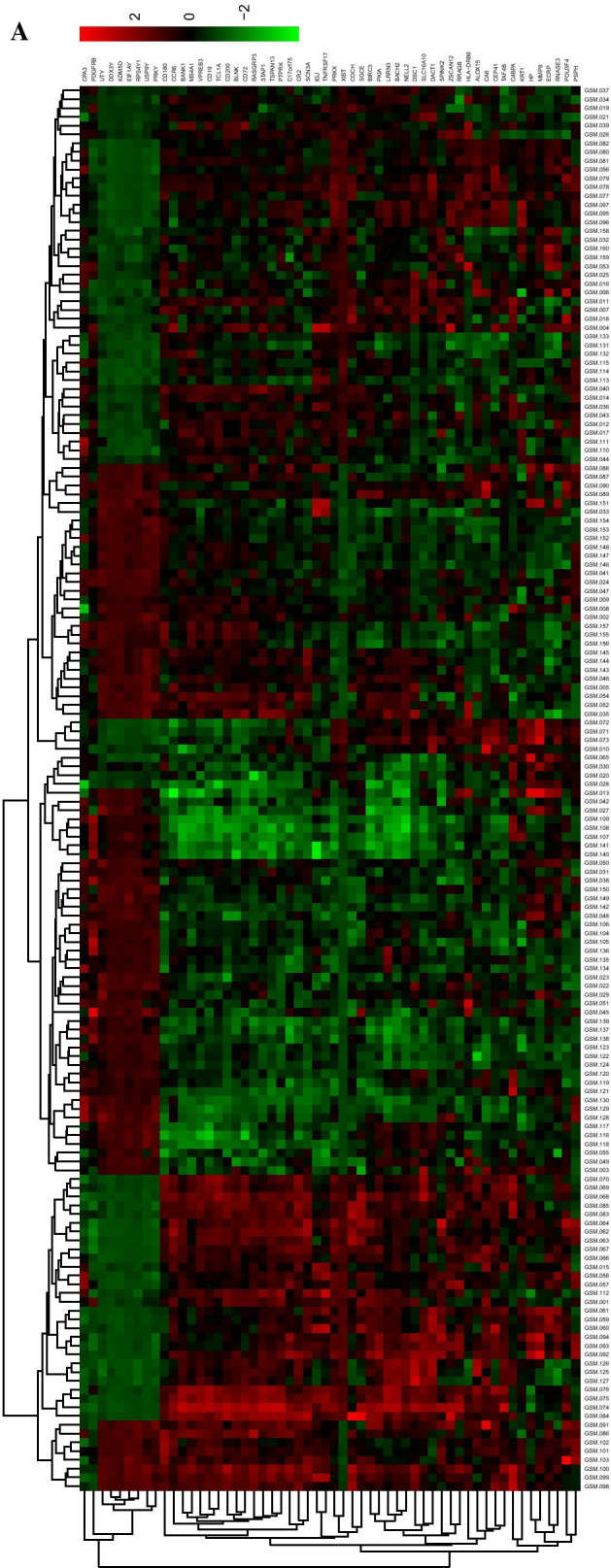

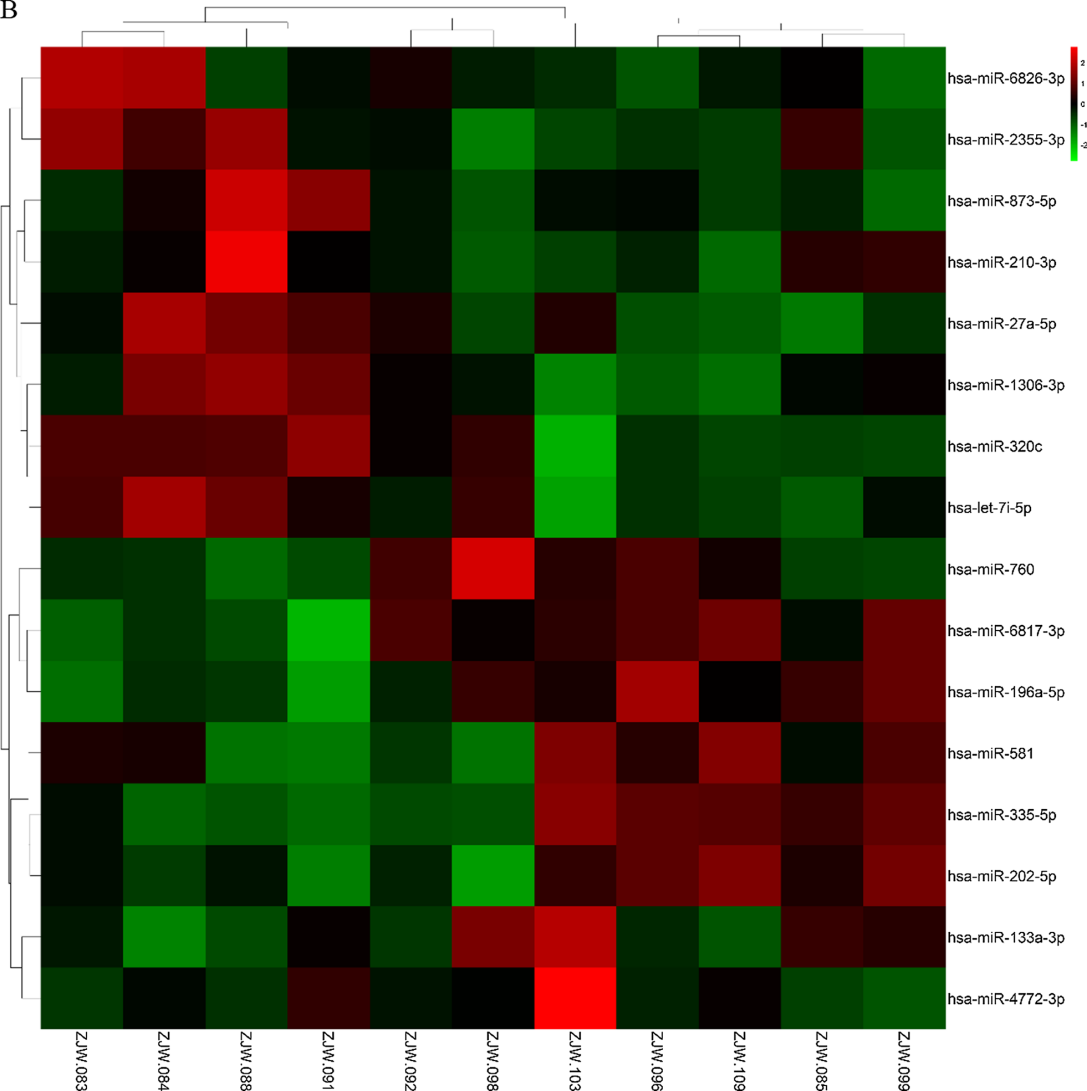
Results from differential expression analysis. **a** The heatmap for differentially expressed genes. (The corresponding number of file names of Fig. 3A was listed in Additional file 1: Table S4) **b** The heatmap for differentially expressed miRNAs (The corresponding number of file names of Fig. 3B was listed in Additional file 1: Table S5). The red represented upregulated genes or miRNAs, the green represented downregulated genes or miRNAs, and the black represented the genes or miRNAs with no significant difference in expression level

Differentially expressed genes were screened by differential expression analysis using limma and DESeq2 packages. The results showed that a total of 56 genes were significantly differentially expressed, among which 47 were up-regulated and 9 were down-regulated (Additional file 1: Table S2). Also, miRNAs were selected by differential expression analysis using DESeq2 packages. There were 16 miRNAs were significantly differentially expressed, among which 8 were up-regulated and 8 were down-regulated (Additional file 1: Table S3). Then, we plotted heatmap to visualize the results by using pheatmap package (Fig. 3a, b).

### MRNA-miRNA co-expression network

**Fig. 4:**
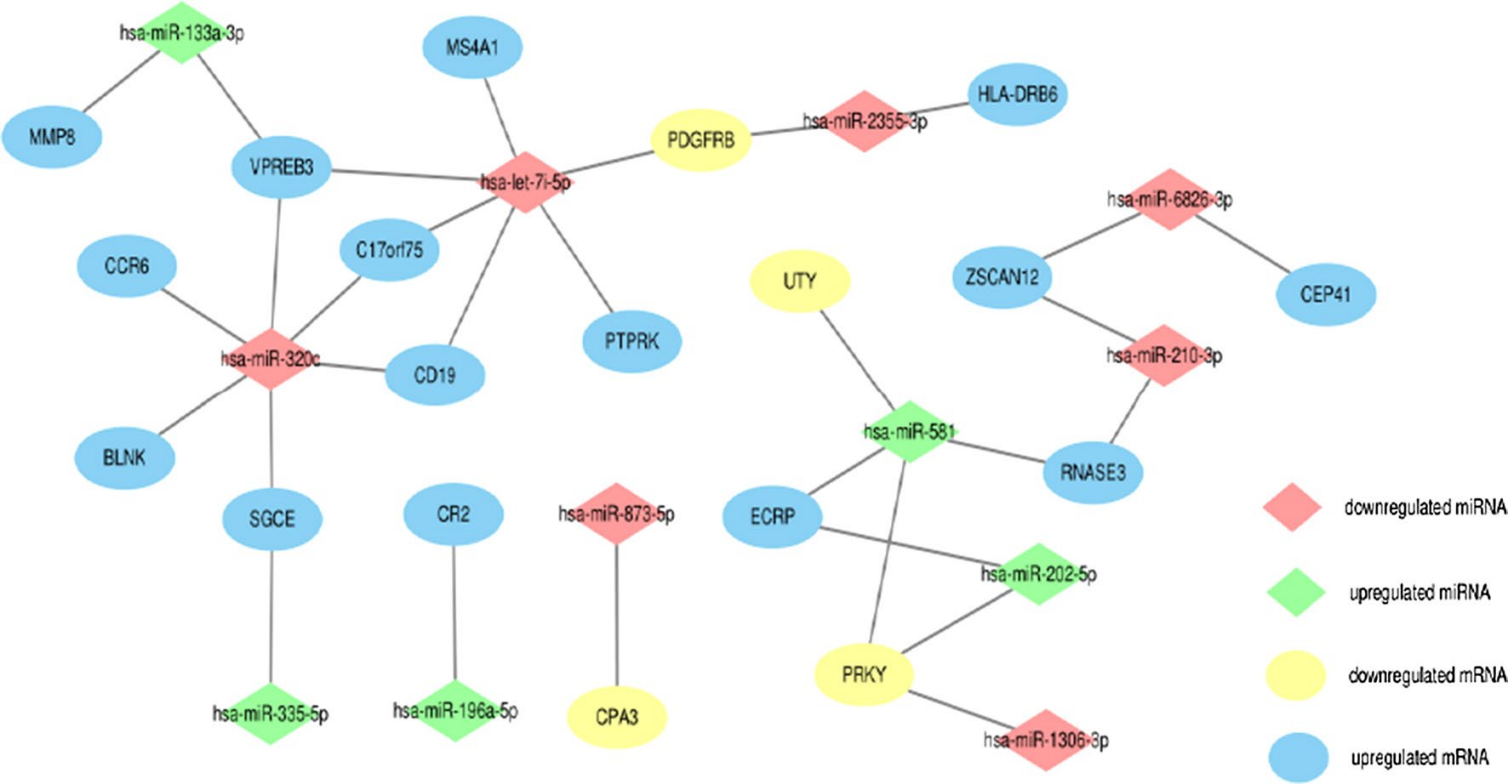
mRNA-miRNA co-expression network

Pearson correlation analysis was performed to explore correlation of differentially expressed mRNA and miRNA, and the co-expression network was visualized, in which 19 mRNA (including 15 upregulated mRNA and 4 downregulated mRNA) and 12 miRNAs (including 5 upregulated miRNAs and 7 downregulated miRNAs) were included (Fig. 4). The let-7i-5p and miR-320c were both correlated to 6 genes.

### GO and KEGG pathway enrichment analysis

**Fig. 5:**
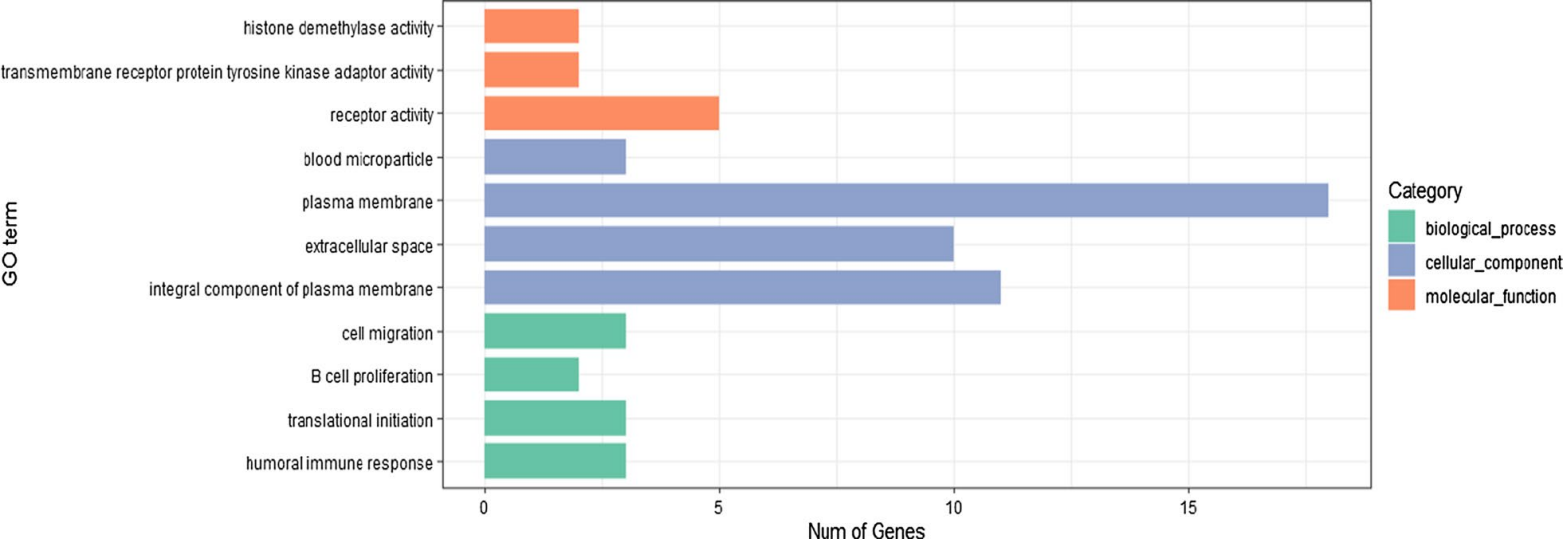
GO analysis of differentially expressed genes

To explore the potential biological functions of differentially expressed genes, GO and KEGG pathway enrichment analyses were performed. The GO results showed that the differentially expressed gene were most likely associated with plasma membrane, extracellular space and receptor activity (Fig. 5). KEGG results revealed that the biological functions of differentially expressed genes included B cell receptor signaling pathway and hematopoietic cell lineage (Table 3).

**Table 3:** Results of KEGG pathway enrichment analysis for differentially expressed genes

Pathway	Gene name	Regulation	*p* value
B cell receptor signaling pathway	*CD72*	Upregulation	< 0.01
	*BLNK*	Upregulation	
	*CD19*	Upregulation	
	*CR2*	Upregulation	
	*RasGRP3*	Upregulation	
hematopoietic cell lineage	*CD19*	Upregulation	0.03
	*MS4A1*	Upregulation	
	*CR2*	Upregulation	

### MRNA-SNP-miRNA interaction network

**Fig. 6:**
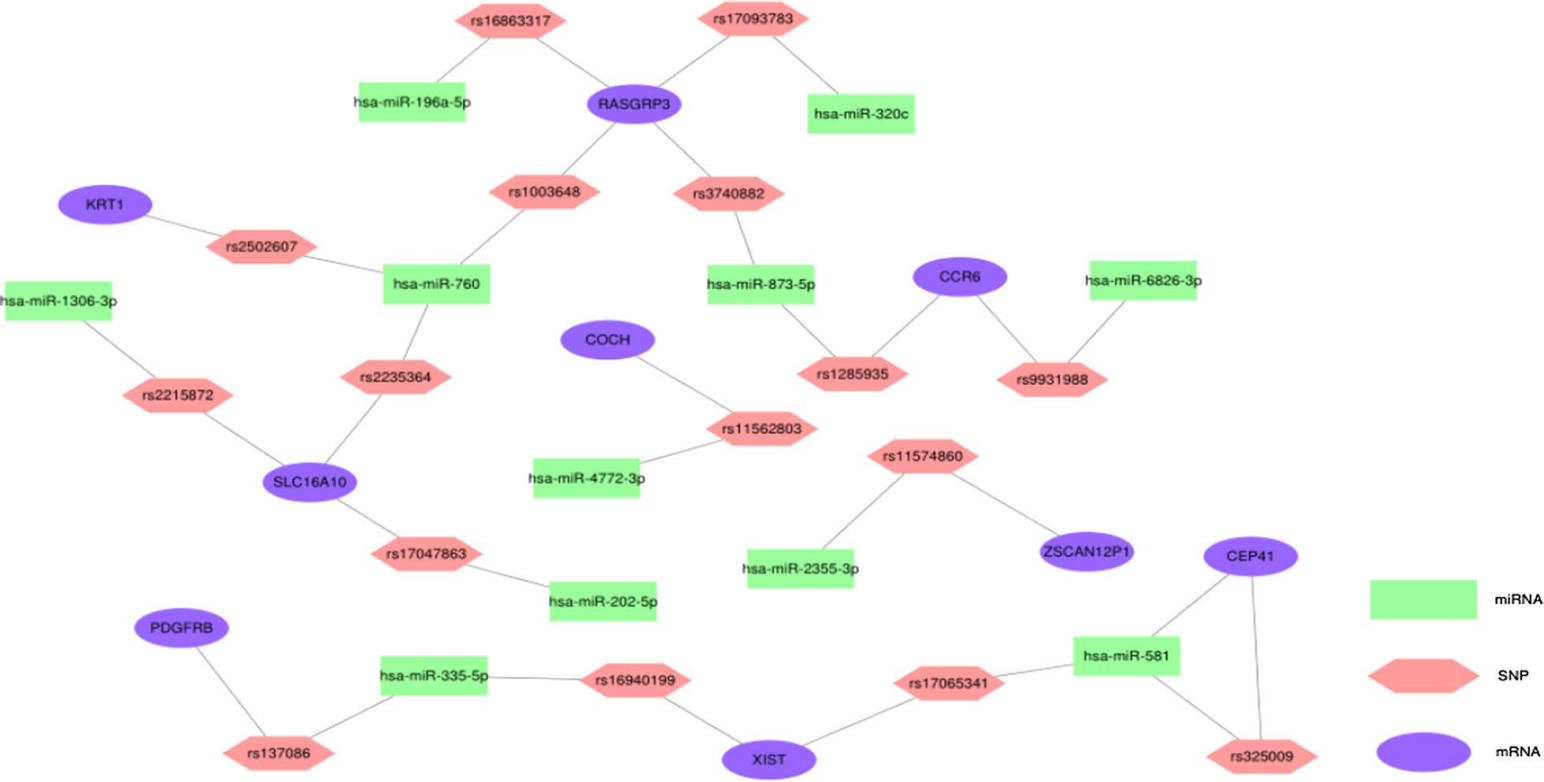
mRNA-SNP-miRNA interaction network

We performed eQTL analysis to explore the association between SNP and mRNA expression level, then we found 60 mRNA and cis-eQTL pairs, and 44,454 mRNA and trans-eQTL pairs.We searched MirSNP and PolymiRTS 3.0 to obtain the SNPs related to differentially expressed miRNAs, eventually we screened 16,018 miRNA and SNP pairs including 16 differentially expressed miRNAs and 15,741 SNPs. Then SNPs related to differentially expressed miRNAs were matched with cis- and trans-eQTL, 16 mRNA-SNP-miRNA trios were obtained by overlapping SNPs. In addition, the effect and binding energy of 8 miRNA and SNP pairs in mRNA-SNP-miRNA trios were obtained (Additional file 1: Table S6). The results showed that rs17093783, rs2235364, rs1285935 and rs11562803 could enhance the binding region, rs2502607, rs325009 and rs11574860 could result in a decrease in the stability of the binding region, and rs17047863 could break the binding region. We also predicted the target mRNA of differentially expressed miRNA, and found that target mRNA of hsa-miR-581 included *CEP41* which was consistent with the mRNA-SNP-miRNA trios. We visualized the relationship between 16 mRNA-SNP-miRNA trios and prediction of differentially expressed miRNA by using software Cytoscape 3.7.2 (Fig. 6).

## Discussion

CVD is the most common cause of death among patients with T2DM, imposing a heavy burden on the economy of individuals and societies. Some studies have investigated the underlying mechanism of CVD and T2DM, and found that miRNA and SNP play important role in the occurrence and development. However, there is still a lack of biomarkers with good sensitivity and specificity for early detection and diagnosis of CVD in T2DM patients. Constructing the mRNA-SNP-miRNA interaction network can help to reveal the underlying correlation between genetic variants and diseases. But there is a lack of miRNA sequencing data of CVD in T2DM patients, leading to few evidence and certain limitations. We combined mRNA, miRNA and SNP data by searching GEO database and conducting miRNA sequencing. Our present study identified 16 mRNA-SNP-miRNA interaction trios, which revealed that SNPs in the binding region between miRNA and mRNA can interfere gene expression. Previous studies have proved that miRNAs play an essential role in the occurrence and development of various diseases [21], and our findings may increase understanding of the underlying mechanism.

In this study, we identified 56 differentially expressed genes and 16 differentially expressed miRNAs between T2DM patients and T2DM patients with CVD. According to mRNA-miRNA co-expression network, let-7i-5p and miR-320c were both related to 6 genes. Previous study showed that let-7i-5p was involved in the regulation of cardiac cell cycle, and inhibition of let-7i-5p may be a potential strategy for cardiac repair after ischemic injury [22]. In addition, downregulated let-7i was observed in dilated cardiomyopathy and low expression of let-7i was associated with poor clinical outcomes of patients with dilated cardiomyopathy [23]. Downregulated let-7i-5p was observed in cardiomyocytes during hypoxia injury and let-7i-5p pathway was used to suppress hypoxia-induced apoptosis and mitochondrial energy metabolism dysfunction in vitro [24]. Taken together, let-7i-5p was demonstrated to be associated with the pathogenesis of CVD, and may play an important role in the pathophysiological process of CVD. Interestingly, the six genes associated with downregulated miR-320 were up-regulated which indicated that miR-320 may bind to complementary sequences of mRNA of these genes to induce mRNA degradation or inhibit translation, thus affecting gene expression.

In our present study, KEGG pathway enrichment analysis indicated significant enrichment in pathways including B cell receptor signaling pathway and hematopoietic cell lineage. Previous studies suggested that compared with healthy people, *CD19* was related to B cell receptor and significantly downregulated in patients with acute myocardial infarction [25]. However, this study showed that *CD19* associated with B cell receptor signaling pathway was upregulated in T2DM patients with CVD, which could attribute to the mechanism of CVD in T2DM patients. Moreover, mutations in the *TET2* that can promote clonal hematopoiesis were associated with an increased risk of atherosclerosis [26]. Interestingly, we found that *TET2* was target gene of 7 differentially expressed miRNAs (miR-133a-3p, miR-6817-3p, miR-873-5p, miR-581, miR-210-3p, miR-202-5p, miR-2355-3p, let-7i-5p, miR-196a-5p, miR-760) in our study which highlighted that the hematopoietic cell lineage and differentially expressed miRNAs may be involved in the progression of CVD in T2DM patients. In addition, KEGG pathway enrichment analysis showed PI3K-Akt signaling pathway was associated with *PDGFRB*, *CD19* and *TCL1A*. Some studies confirmed that PI3K-Akt signaling pathway plays an important role in the pathophysiology of vascular diseases [27]. Activated PI3K-Akt signaling pathway has been proven to improve insulin sensitivity, regulate glucose and lipid metabolism, and protect vascular endothelial cells [28]. In addition, activating IRS/PI3K/Akt pathway activity may play an anti-atherosclerotic role [29]. Interestingly, KEGG pathway enrichment analysis indicated that linoleic acid metabolism was related to *ALOX15*, and a growing number of studies reported that linoleic acid was associated with the prevention of T2DM and CVD [30, 31]. Therefore, we can reasonably speculate that PI3K-Akt signaling pathway and linoleic acid metabolism may play a role in the pathogenesis of T2DM complicated with CVD.

MRNA-SNP-miRNA interaction network included 16 SNPs, 9 mRNA and 11 miRNAs, but only miR-581, rs325009 and *CEP41* were related to each other. Previous studies suggested that *CEP41* was a new regulator for angiogenesis which promoted angiogenesis through the HIF1A-Aurka-VEGF pathway and was involved in endothelial cell migration which was involved in the pathogenesis of CVD [32]. Our study showed that *RASGRP3* was associated with 4 SNPs in the mRNA-SNP-miRNA interaction network, and each intermediated one pairs of miRNA-mRNA correlations, which indicated that *RASGRP3* may play a key role in the underlying mechanism. Paramjeet and colleagues observed that *RASGRP3* could affect the role of endothelial cells in angiogenesis in diabetic mice by mediating endothelial cell signal transduction [33]. We further explored functions of the rest of mRNA in the mRNA-SNP-miRNA interaction network, and found that *KRT1* might be associated with CVD. Gao et al. found that inhibiting *KRT1* can activate Notch signaling pathway, thereby inhibiting the inflammatory response and endoplasmic reticulum stress of vascular endothelial cells in coronary atherosclerosis [34]. Furthermore, it has been reported that inhibition of *KRT1* expression can improve myocardial ischemia–reperfusion injury by activating Notch signaling pathway [35].

Although previous studies have shown that miRNA-related SNPs play a role in the pathogenesis of T2DM complicated with CVD, there is a lack of relevant researches that combine mRNA, miRNA and SNP for analysis. We searched GEO database and found that there are few studies involving miRNA sequencing for T2DM patients and T2DM patients with CVD. Hence, we recruited six diabetes patients and five diabetes patients with ischemic heart disease from communities in Beijing to obtain their miRNA expression profile. Previous studies suggested that miR-196a-5p and miR-202-5p may play a role in the pathogenesis of diabetes and its cardiovascular complications. Omer et al. revealed that compared with normal group, miR-196a-5p was downregulated in CVD group without statistically significance, and they indicated that miR-196a-5p may be a potential biomarker for the diagnosis of coronary artery disease and acute coronary syndrome [36]. However, our present study suggested that miR-196a-5p was differentially upregulated in CVD group, which was inconsistent with previous study. We attributed the difference to the limited sample size, and studies with large samples are needed to verify the results. In addition, up-regulated miR-202-5p was found to have a protective effect on the heart of mice with myocardial ischemia–reperfusion injury [37]. But our present study identified that miR-202-5p was differentially upregulated in CVD group, and we considered that it was due to the different species in two studies which weakened the comparability.

We successfully constructed the mRNA-SNP-miRNA interaction network to visualize the relationships between mRNAs, SNP and miRNAs. However, there was no statistical significance in Pearson correlation analysis of mRNA and miRNA connected by overlapping SNPs in the mRNA-SNP-miRNA interaction network. Considering this study, the reasons may be associated with limitations in this study. First, the sample size of miRNA is so small that its results are not representative enough, and future studies including larger samples are required; second, mRNA and miRNA expression data were obtained from different samples, resulting in bias although we searched eligible databases by defining the CVD clearly and setting covariates to eliminate the bias, and miRNA, mRNA and SNP data from same experiment are needed to provide more convincing research results. Moreover, some miRNAs cannot inhibit mRNA expression but can inhibit protein translation. Hence, the co-expression analysis of mRNA and miRNA was not statistically significant, which did not indicate that there was no regulatory relationship between them. Further study on the significance of miRNA and mRNA at protein level is required.

## Conclusion

We demonstrated mRNA-SNP-miRNA interaction network and identified important pathways contributing to metabolic cardiovascular disease. These results suggested that downregulated let-7i-5p, and upregulated *RASGRP3*, *KRT1* and *CEP41* may play crucial roles in molecular mechanisms underlying the initiation and development of CVD in T2DM patients. However, further studies regarding the role of these genes and miRNAs in progression of CVD in T2DM patients are required.

## Supplementary Information

**Additional file 1: Figure S1**. The normalized unscaled standard errors boxplot from quality evaluation analysis for dataset GSE66175. **Figure S2**. The RNA degradation plot from quality evaluation analysis for dataset GSE66175. **Figure S3**. KEGG pathway enrichment analysis for differentially expressed genes. **Table S1**. Sample IDs. **Table S2**. Top 10 mRNA in absolute value of log2(fold change). **Table S3**. Top 10 miRNA in absolute value of log2(fold change). **Table S4**. The corresponding number of file names in Fig 3A. **Table S5**. The corresponding number of file names in Fig. 3B. **Table S6**. The relationship between miRNA and SNP in mRNA-SNP-miRNA trios.

## Data Availability

Microarray datasets (GSE90074, GSE66175, and GSE90073) for this study are openly available in Gene Expression Omnibus database at https://www.ncbi.nlm.nih.gov/geo/query/acc.cgi?acc = GSE90074, https://www.ncbi.nlm.nih.gov/geo/query/acc.cgi?acc = GSE66175, and https://www.ncbi.nlm.nih.gov/geo/query/acc.cgi?acc = GSE90073, respectively (last accessed on 23 Jan 2020). The datasets of miRNA used during the current study are available from the corresponding author on reasonable request.

## Abbreviations

CVD: Cardiovascular diseases
T2DM: Type 2 diabetes mellitus
miRNA: MicroRNA
SNP: Single nucleotide polymorphisms
GEO: Gene expression omnibus
GO: Gene ontology
KEGG: Kyoto encyclopedia of genes and genomes
MRE: MiRNA recognition element
GWAS: Genome-wide association studies
eQTL: Genome-wide expression quantitative trait locus
