# Supplementary material for: Identification of biomarkers associated with metabolic cardiovascular disease using mRNA-SNP-miRNA regulatory network analysis
Source: BMC Cardiovasc Disord. 2021 Jul 23;21:351. doi: 10.1186/s12872-021-02166-4 (PMC8305867; doi:10.1186/s12872-021-02166-4)
Supplement: Supplementary file 1 — Additional file 1: Figure S1. The normalized unscaled standard errors boxplot from quality evaluation analysis for dataset GSE66175. Figure S2. The RNA degradation plot from quality evaluation analysis for dataset GSE66175. Figure S3. KEGG pathway enrichment analysis for differentially expressed genes. Table S1. Sample IDs. Table S2. Top 10 mRNA in absolute value of log2(fold change). Table S3. Top 10 miRNA in absolute value of log2(fold change). Table S4. The corresponding number of file names in Fig 3A. Table S5. The corresponding number of file names in Fig. 3B. Table S6. The relationship between miRNA and SNP in mRNA-SNP-miRNA trios. [file 12872_2021_2166_MOESM1_ESM.docx]

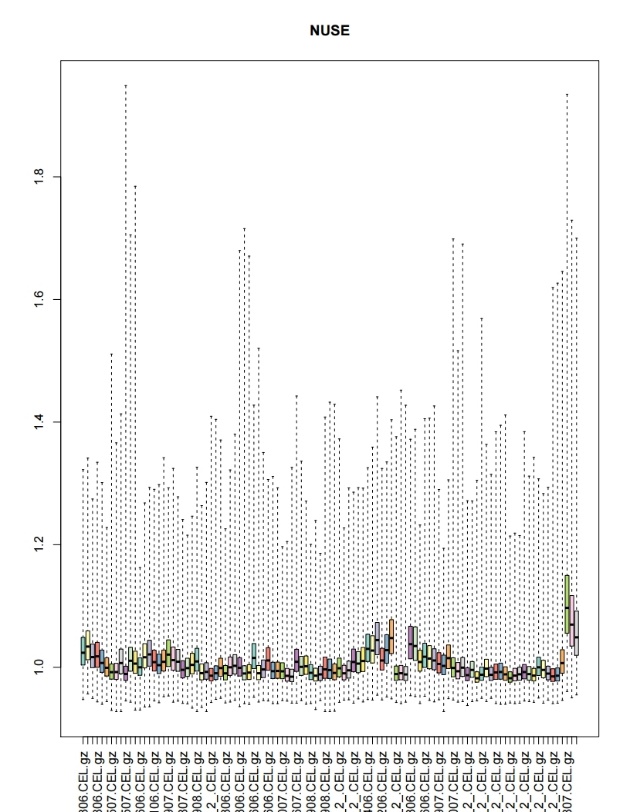


**Supplementary Fig. 1** The normalized unscaled standard errors boxplot from quality evaluation analysis for dataset GSE66175.


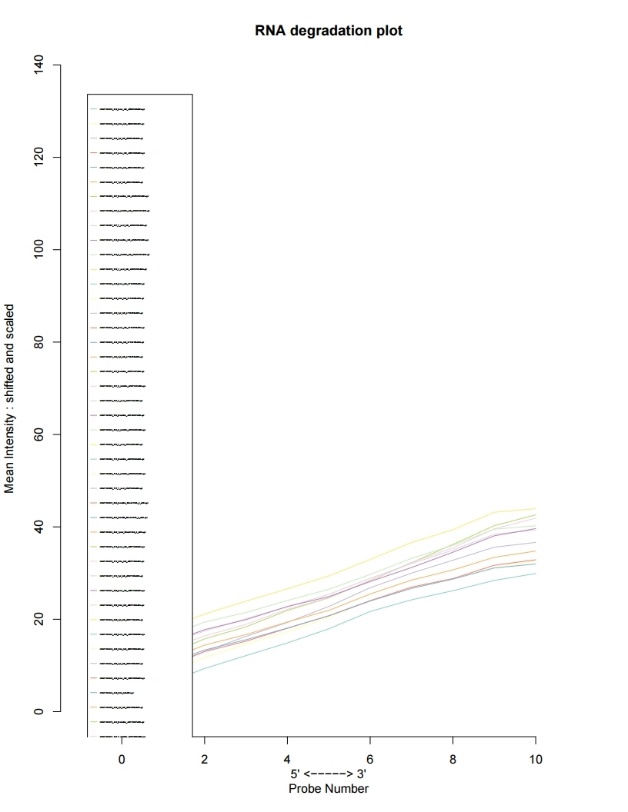


**Supplementary Fig. 2** The RNA degradation plot from quality evaluation analysis for dataset GSE66175.


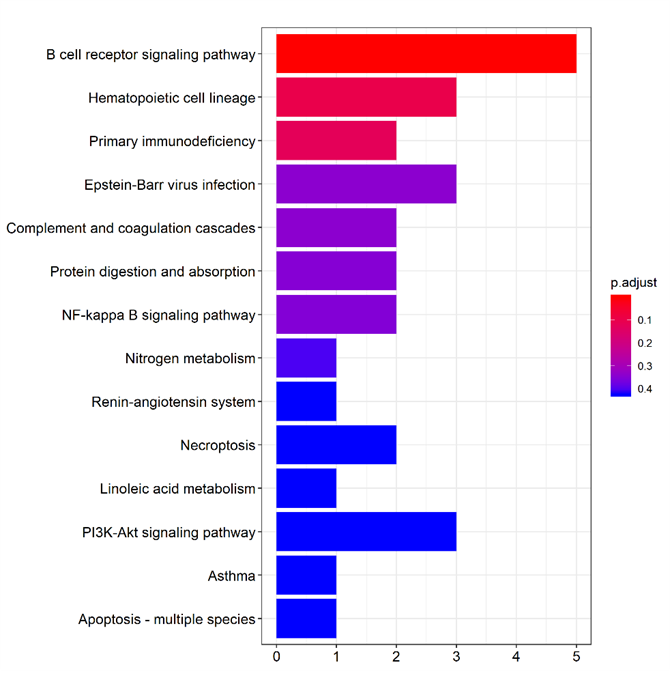


**Supplementary Fig. 3** KEGG pathway enrichment analysis for differentially expressed genes.

**Supplementary Table 1.** **Sample IDs**

| **GSE** | **Disease** | **GSM** |
| --- | --- | --- |
| **GSE90074** | **T2DM** | **GSM2397158 GSM2397161 GSM2397180**  **GSM2397182 GSM2397189 GSM2397208**  **GSM2397209 GSM2397223 GSM2397224**  **GSM2397233 GSM2397249 GSM2397252**  **GSM2397263 GSM2397277 GSM2397279**  **GSM2397283 GSM2397286 GSM2397159**  **GSM2397168 GSM2397170 GSM2397177**  **GSM2397179 GSM2397184 GSM2397185**  **GSM2397191 GSM2397193** |
|  | **T2DM+CVD** | **GSM2397210 GSM2397211 GSM2397212**  **GSM2397215 GSM2397220 GSM2397222**  **GSM2397226 GSM2397229 GSM2397232**  **GSM2397240 GSM2397241 GSM2397242**  **GSM2397250 GSM2397262 GSM2397265**  **GSM2397266 GSM2397268 GSM2397269**  **GSM2397271 GSM2397273 GSM2397274**  **GSM2397275 GSM2397280 GSM2397285**  **GSM2397287 GSM2397288 GSM2397293**  **GSM2397296 GSM2397298** |
| **GSE66175** | **T2DM** | **GSM1123265 GSM1123266 GSM1123267**  **GSM1123289 GSM1123290 GSM1123291**  **GSM1123328 GSM1123329 GSM1123330**  **GSM1123364 GSM1123365 GSM1123366**  **GSM1123397 GSM1123398 GSM1123399**  **GSM1123415 GSM1123416 GSM1123417**  **GSM1123421 GSM1123422 GSM1123423**  **GSM1123448 GSM1123449 GSM1123450**  **GSM1123478 GSM1123479 GSM1123480**  **GSM1123490 GSM1123491 GSM1123492**  **GSM1616305 GSM1616306 GSM1616307**  **GSM1616311 GSM1616312 GSM1616313**  **GSM1616323 GSM1616324 GSM1616325**  **GSM1616341 GSM1616342 GSM1616343**  **GSM1616356 GSM1616357 GSM1616358**  **GSM1616371 GSM1616372 GSM1616373** |
|  | **T2DM+CVD** | **GSM1123235 GSM1123236 GSM1123237 GSM1123244 GSM1123245 GSM1123246 GSM1123274 GSM1123275 GSM1123276 GSM1123295 GSM1123296 GSM1123297 GSM1123313 GSM1123314 GSM1123315 GSM1123442 GSM1123443 GSM1123444 GSM1123508 GSM1123509 GSM1123510 GSM1123550 GSM1123551 GSM1123552 GSM1123583 GSM1123584 GSM1123585 GSM1123601 GSM1123602 GSM1123603 GSM1616278 GSM1616279 GSM1616280 GSM1616290 GSM1616291 GSM1616292 GSM1616308 GSM1616309 GSM1616310 GSM1616320 GSM1616321 GSM1616322 GSM1616326 GSM1616327 GSM1616328 GSM1616350 GSM1616351 GSM1616352 GSM1616362 GSM1616363 GSM1616364 GSM1616368 GSM1616369 GSM1616370 GSM1616377 GSM1616378 GSM1616379** |
| **GSE90073** | **T2DM** | **GSM2397052 GSM2397055 GSM2397070 GSM2397072 GSM2397077 GSM2397092 GSM2397093 GSM2397110 GSM2397121 GSM2397124 GSM2397135 GSM2397144 GSM2397145** |
|  | **T2DM+CVD** | **GSM2397053 GSM2397060 GSM2397061 GSM2397067 GSM2397069 GSM2397080 GSM2397094 GSM2397095 GSM2397098 GSM2397102 GSM2397103 GSM2397106 GSM2397109 GSM2397115 GSM2397116 GSM2397122 GSM2397134 GSM2397137 GSM2397139 GSM2397140 GSM2397141 GSM2397142 GSM2397146 GSM2397149 GSM2397150** |

**Supplementary Table 2.** Top 10 mRNA in absolute value of log2(fold change)

| mRNA | *P*-value | LogFC | regulation |
| --- | --- | --- | --- |
| *XIST* | <0.001 | 3.52 | upregulation |
| *DDX3Y* | <0.001 | -2.21 | downregulation |
| *KDM5D* | <0.001 | -2.04 | downregulation |
| *RPS4Y1* | <0.001 | -2.02 | downregulation |
| *EIF1AY* | <0.001 | -1.49 | downregulation |
| *USP9Y* | <0.001 | -1.18 | downregulation |
| *HLA-DRB6* | <0.001 | 0.95 | upregulation |
| *RASGRP3* | <0.001 | 0.91 | upregulation |
| *IGJ* | <0.001 | 0.89 | upregulation |
| *TSPAN13* | <0.001 | 0.89 | upregulation |

**Supplementary Table 3.** Top 10 miRNA in absolute value of log2(fold change)

| miRNA | *P*-value | LogFC | regulation |
| --- | --- | --- | --- |
| miR-6826-3p | 0.0173 | -2.05 | downregulation |
| miR-873-5p | 0.0237 | -1.93 | downregulation |
| miR-760 | 0.0426 | 1.67 | upregulation |
| miR-196a-5p | 0.0373 | 1.45 | upregulation |
| miR-202-5p | 0.0228 | 1.36 | upregulation |
| miR-4772-3p | 0.0462 | 1.36 | upregulation |
| miR-133a-3p | 0.0220 | 1.28 | upregulation |
| miR-2355-3p | 0.0123 | -1.27 | downregulation |
| miR-1306-3p | 0.0056 | -1.01 | downregulation |
| miR-335-5p | 0.0048 | 0.99 | upregulation |

**Supplementary Table 4.** The corresponding number of file names in Fig 3A

| File name | Number |
| --- | --- |
| GSM2397158 | GSM-001 |
| GSM2397161 | GSM-002 |
| GSM2397180 | GSM-003 |
| GSM2397182 | GSM-004 |
| GSM2397189 | GSM-005 |
| GSM2397208 | GSM-006 |
| GSM2397209 | GSM-007 |
| GSM2397223 | GSM-008 |
| GSM2397224 | GSM-009 |
| GSM2397233 | GSM-010 |
| GSM2397249 | GSM-011 |
| GSM2397252 | GSM-012 |
| GSM2397263 | GSM-013 |
| GSM2397277 | GSM-014 |
| GSM2397279 | GSM-015 |
| GSM2397283 | GSM-016 |
| GSM2397286 | GSM-017 |
| GSM2397159 | GSM-018 |
| GSM2397168 | GSM-019 |
| GSM2397170 | GSM-020 |
| GSM2397177 | GSM-021 |
| GSM2397179 | GSM-022 |
| GSM2397184 | GSM-023 |
| GSM2397185 | GSM-024 |
| GSM2397191 | GSM-025 |
| GSM2397193 | GSM-026 |
| GSM2397210 | GSM-027 |
| GSM2397211 | GSM-028 |
| GSM2397212 | GSM-029 |
| GSM2397215 | GSM-030 |
| GSM2397220 | GSM-031 |
| GSM2397222 | GSM-032 |
| GSM2397226 | GSM-033 |
| GSM2397229 | GSM-034 |
| GSM2397232 | GSM-035 |
| GSM2397240 | GSM-036 |
| GSM2397241 | GSM-037 |
| GSM2397242 | GSM-038 |
| GSM2397250 | GSM-039 |
| GSM2397262 | GSM-040 |
| GSM2397265 | GSM-041 |
| GSM2397266 | GSM-042 |
| GSM2397268 | GSM-043 |
| GSM2397269 | GSM-044 |
| GSM2397271 | GSM-045 |
| GSM2397273 | GSM-046 |
| GSM2397274 | GSM-047 |
| GSM2397275 | GSM-048 |
| GSM2397280 | GSM-049 |
| GSM2397285 | GSM-050 |
| GSM2397287 | GSM-051 |
| GSM2397288 | GSM-052 |
| GSM2397293 | GSM-053 |
| GSM2397296 | GSM-054 |
| GSM2397298 | GSM-055 |
| GSM1123265_307_Baseline_GC_JW052507.CEL | GSM-056 |
| GSM1123266_307_3_months_GC_JW052507.CEL | GSM-057 |
| GSM1123267_307_1_year_GC_JW052507.CEL | GSM-058 |
| GSM1123289_428_base_GC_LF102606.CEL | GSM-059 |
| GSM1123290_428_3mo_GC_LF102606.CEL | GSM-060 |
| GSM1123291_428_1yr_GC_LF102606.CEL | GSM-061 |
| GSM1123328_589_Baseline_JW122807.CEL | GSM-062 |
| GSM1123329_589_3_months_JW122807.CEL | GSM-063 |
| GSM1123330_589_1_year_JW122807.CEL | GSM-064 |
| GSM1123364_751_Baseline_JW070908.CEL | GSM-065 |
| GSM1123365_751_3_months_JW070908.CEL | GSM-066 |
| GSM1123366_751_1_year_JW070908.CEL | GSM-067 |
| GSM1123397_884_baseline_HG.U133A_2_.CEL | GSM-068 |
| GSM1123398_884_3month_HG.U133A_2_.CEL | GSM-069 |
| GSM1123399_884_1year_HG.U133A_2_.CEL | GSM-070 |
| GSM1123415_374_base_GC_JH071306.CEL | GSM-071 |
| GSM1123416_374_3mo_GC_JH071306.CEL | GSM-072 |
| GSM1123417_374_1yr_GC_JH071306.CEL | GSM-073 |
| GSM1123421_380_base_GC_JH071306.CEL | GSM-074 |
| GSM1123422_380_3mo_GC_JH071306.CEL | GSM-075 |
| GSM1123423_380_1yr_GC_JH071306.CEL | GSM-076 |
| GSM1123448_440_base_GC_JH072606.CEL | GSM-077 |
| GSM1123449_440_3mo_GC.CEL | GSM-078 |
| GSM1123450_440_1yr_GC_JH072606.CEL | GSM-079 |
| GSM1123478_492_Baseline_JW113007.CEL | GSM-080 |
| GSM1123479_492_3_months_JW113007.CEL | GSM-081 |
| GSM1123480_492_1_year_JW113007.CEL | GSM-082 |
| GSM1123490_496_Baseline_JW110607.CEL | GSM-083 |
| GSM1123491_496_3_months_JW110607.CEL | GSM-084 |
| GSM1123492_496_1_year_JW110607.CEL | GSM-085 |
| GSM1616305_414_1year_HG.U133A_2_.CEL | GSM-086 |
| GSM1616306_414_3month_HG.U133A_2_.CEL | GSM-087 |
| GSM1616307_414_baseline_HG.U133A_2_.CEL | GSM-088 |
| GSM1616311_429_1yr_GC_JH062606.CEL | GSM-089 |
| GSM1616312_429_3mo_GC_JH062606.CEL | GSM-090 |
| GSM1616313_429_base_GC_JH062606.CEL | GSM-091 |
| GSM1616323_579_1_year_JW_122007.CEL | GSM-092 |
| GSM1616324_579_3_months_JW_122007.CEL | GSM-093 |
| GSM1616325_579_Baseline_JW_122007.CEL | GSM-094 |
| GSM1616341_749_1year_HG.U133A_2_.CEL | GSM-095 |
| GSM1616342_749_3month_HG.U133A_2_.CEL | GSM-096 |
| GSM1616343_749_baseline_HG.U133A_2_.CEL | GSM-097 |
| GSM1616356_880_1year_HG.U133A_2_.CEL | GSM-098 |
| GSM1616357_880_3month_HG.U133A_2_.CEL | GSM-099 |
| GSM1616358_880_baseline_HG.U133A_2_.CEL | GSM-100 |
| GSM1616371_933_1year_HG.U133A_2_.CEL | GSM-101 |
| GSM1616372_933_3month_HG.U133A_2_.CEL | GSM-102 |
| GSM1616373_933_baseline_HG.U133A_2_.CEL | GSM-103 |
| GSM1123235_292_base_GC_JH062306.CEL | GSM-104 |
| GSM1123236_292_3mo_GC_JH062306.CEL | GSM-105 |
| GSM1123237_292_1yr_GC_JH062306.CEL | GSM-106 |
| GSM1123244_297_base_GC_JH091806.CEL | GSM-107 |
| GSM1123245_297_3mo_GC_JH091806.CEL | GSM-108 |
| GSM1123246_297_1yr_GC_JH091806.CEL | GSM-109 |
| GSM1123274_310_Baseline_GC_JW052507.CEL | GSM-110 |
| GSM1123275_310_3_months_GC_JW052507.CEL | GSM-111 |
| GSM1123276_310_1_year_GC_JW052507.CEL | GSM-112 |
| GSM1123295_432_base_GC_LF103106.CEL | GSM-113 |
| GSM1123296_432_3mo_GC_LF103106.CEL | GSM-114 |
| GSM1123297_432_1yr_GC_LF103106.CEL | GSM-115 |
| GSM1123313_537_Baseline_JW071907.CEL | GSM-116 |
| GSM1123314_537_3_months_JW071907.CEL | GSM-117 |
| GSM1123315_537_1_year_JW071907.CEL | GSM-118 |
| GSM1123442_400_base_GC_JH072606.CEL | GSM-119 |
| GSM1123443_400_3mo_GC_JH072606.CEL | GSM-120 |
| GSM1123444_400_1yr_GC_JH072606.CEL | GSM-121 |
| GSM1123508_554_Baseline_JW070908.CEL | GSM-122 |
| GSM1123509_554_3_months_JW062708.CEL | GSM-123 |
| GSM1123510_554_1_year_JW062708.CEL | GSM-124 |
| GSM1123550_690_Baseline_JW070908.CEL | GSM-125 |
| GSM1123551_690_3_months_JW070908.CEL | GSM-126 |
| GSM1123552_690_1_year_JW070908.CEL | GSM-127 |
| GSM1123583_771_baseline_HG.U133A_2_.CEL | GSM-128 |
| GSM1123584_771_3month_HG.U133A_2_.CEL | GSM-129 |
| GSM1123585_771_1year_HG.U133A_2_.CEL | GSM-130 |
| GSM1123601_942_baseline_HG.U133A_2_.CEL | GSM-131 |
| GSM1123602_942_3month_HG.U133A_2_.CEL | GSM-132 |
| GSM1123603_942_1year_HG.U133A_2_.CEL | GSM-133 |
| GSM1616278_287_1yr_GC_JH061406.CEL | GSM-134 |
| GSM1616279_287_3mo_GC_JH061406.CEL | GSM-135 |
| GSM1616280_287_base_GC_JH061406.CEL | GSM-136 |
| GSM1616290_350_1yr_GC_JH062206.CEL | GSM-137 |
| GSM1616291_350_3mo_GC_JH062206.CEL | GSM-138 |
| GSM1616292_350_base_GC_JH062206.CEL | GSM-139 |
| GSM1616308_427_1yr_GC_JH062306.CEL | GSM-140 |
| GSM1616309_427_3mo_GC_JH062306.CEL | GSM-141 |
| GSM1616310_427_base_GC_JH062306.CEL | GSM-142 |
| GSM1616320_538_1_year_JW_071907.CEL | GSM-143 |
| GSM1616321_538_3_months_JW_071907.CEL | GSM-144 |
| GSM1616322_538_Baseline_JW_071907.CEL | GSM-145 |
| GSM1616326_666_1year_HG.U133A_2_.CEL | GSM-146 |
| GSM1616327_666_3month_HG.U133A_2_.CEL | GSM-147 |
| GSM1616328_666_baseline_HG.U133A_2_.CEL | GSM-148 |
| GSM1616350_812_1year_HG.U133A_2_.CEL | GSM-149 |
| GSM1616351_812_3month_HG.U133A_2_.CEL | GSM-150 |
| GSM1616352_812_baseline_HG.U133A_2_.CEL | GSM-151 |
| GSM1616362_885_1year_HG.U133A_2_.CEL | GSM-152 |
| GSM1616363_885_3month_HG.U133A_2_.CEL | GSM-153 |
| GSM1616364_885_baseline_HG.U133A_2_.CEL | GSM-154 |
| GSM1616368_919_1year_HG.U133A_2_.CEL | GSM-155 |
| GSM1616369_919_3month_HG.U133A_2_.CEL | GSM-156 |
| GSM1616370_919_baseline_HG.U133A_2_.CEL | GSM-157 |
| GSM1616377_CVD_300_Baseline_032807.CEL | GSM-158 |
| GSM1616378_CVD_300_1_year_032807.CEL | GSM-159 |
| GSM1616379_CVD_300_3_months_032807.CEL | GSM-160 |

**Supplementary Table 5.** The corresponding number of file names in Fig. 3B

| File name | Number |
| --- | --- |
| DM-01 | ZJW-083 |
| DM-02 | ZJW-084 |
| DM-03 | ZJW-085 |
| DM-04 | ZJW-088 |
| DM-05 | ZJW-091 |
| DM-06 | ZJW-092 |
| DM+CVD-01 | ZJW-096 |
| DM+CVD-02 | ZJW-098 |
| DM+CVD-03 | ZJW-099 |
| DM+CVD-04 | ZJW-103 |
| DM+CVD-05 | ZJW-109 |

**Supplementary Table 6.** The relationship between miRNA and SNP in mRNA-SNP-miRNA trios

| miRNA | SNP | mirSVR | Effect | Allele | Score | Energy | Conservation |
| --- | --- | --- | --- | --- | --- | --- | --- |
| hsa-miR-320c | rs17093783 | -1.04 | enhance | A | 149 | -13.23 | 0.993 |
| hsa-miR-760 | rs2502607 | -0.257 | decrease | C | 159 | -25.37 | 0.007 |
| hsa-miR-760 | rs2235364 | -0.001 | enhance | G | 163 | -25.22 | 0.002 |
| hsa-miR-581 | rs325009 | -0.056 | decrease | A | 158 | -15.22 | 0 |
| hsa-miR-873-5p | rs1285935 | -0.299 | enhance | G | 147 | -17.49 | 0 |
| hsa-miR-4772-3p | rs11562803 | - | enhance | G | 142 | -15.68 | 0.001 |
| hsa-miR-202-5p | rs17047863 | -0.687 | break | C | 157 | -20.01 | 0.077 |
| hsa-miR-2355-3p | rs11574860 | - | decrease | G | 143 | -11.58 | 0.012 |
